# Onchocerciasis in the Ntui Health District of Cameroon: epidemiological, entomological and parasitological findings in relation to elimination prospects

**DOI:** 10.1186/s13071-022-05585-0

**Published:** 2022-11-28

**Authors:** Joseph Nelson Siewe Fodjo, Leonard Ngarka, Wepnyu Yembe Njamnshi, Peter Ayuk Enyong, Anne-Cécile Zoung-Kanyi Bissek, Alfred Kongnyu Njamnshi

**Affiliations:** 1 Brain Research Africa Initiative (BRAIN), Yaoundé, Cameroon; 2 Neurology Department, Central Hospital Yaoundé, Yaoundé, Cameroon; 3 Neuroscience Lab, Faculty of Medicine and Biomedical Sciences, The University of Yaoundé I, Yaoundé, Cameroon; 4 Ministry of Public Health, Division of Health Operations Research, Yaoundé, Cameroon; 5 Research Foundation in Tropical Diseases and Environment (REFOTDE), Buea, Cameroon

**Keywords:** *Onchocerca volvulus*, Ivermectin, Black flies, Biting rates, Transmission potentials, Cameroon

## Abstract

**Background:**

Despite decades of community-directed treatment with ivermectin (CDTI), onchocerciasis transmission persists in Cameroon and has been associated with increased risk for epilepsy in endemic communities. We investigated the onchocerciasis situation in the Ntui Health District (a known onchocerciasis focus in Cameroon where the Sanaga River constitutes the main source of black fly vectors) using parasitological, entomological and serological parameters.

**Methods:**

In July 2021, community-based surveys were conducted in four villages (Essougli, Nachtigal, Ndjame and Ndowe). Onchocerciasis was diagnosed via microscopic examination of skin snips. Using rapid diagnostic tests, we screened children aged 3–6 years for Ov16 antibodies as a proxy for recent onchocerciasis transmission. Monthly black fly biting rates were obtained from the two riverside villages (Nachtigal and Essougli) for 12 consecutive months (July 2021 to June 2022) using the human landing catch technique. Some black flies were dissected each month to check for infection.

**Results:**

Overall, 460 participants were recruited; mean age was 32.1 (range: 3–85) years with 248 (53.9%) being males. Among skin snipped participants (*n* = 425), onchocerciasis prevalence was 14.6%. Participants with epilepsy (*n* = 25) were more often skin snip positive (45.8% vs 12.7%; *P* < 0.001) and had higher microfilarial loads (9.2 ± 22.0 vs 0.7 ± 3.5 microfilariae/skin snip; *P* < 0.001) compared to their peers without epilepsy. Eight (6.5%) of the 123 tested children were Ov16 seropositive. The breeding sites we investigated along the Sanaga River during the current study harbored fewer vectors (annual biting rates reaching 530,322 vs 606,370 in the Mbam River) and exhibited lower black fly infection rates (annual transmission potentials reaching 1479 vs 4488 in the Mbam River) when compared to recent entomological reports in Cameroon.

**Conclusion:**

Despite substantial biting rates, black fly infection rates (by microscopy) in the Ntui Health District were rather low resulting in overall low transmission potentials in study villages. Thanks to CDTI, *O. volvulus* infection in both humans and insects is on the decrease. However, there is evidence that *O. volvulus* is still endemic in these communities. Reducing the vector population will further accelerate onchocerciasis elimination prospects.

**Graphical Abstract:**

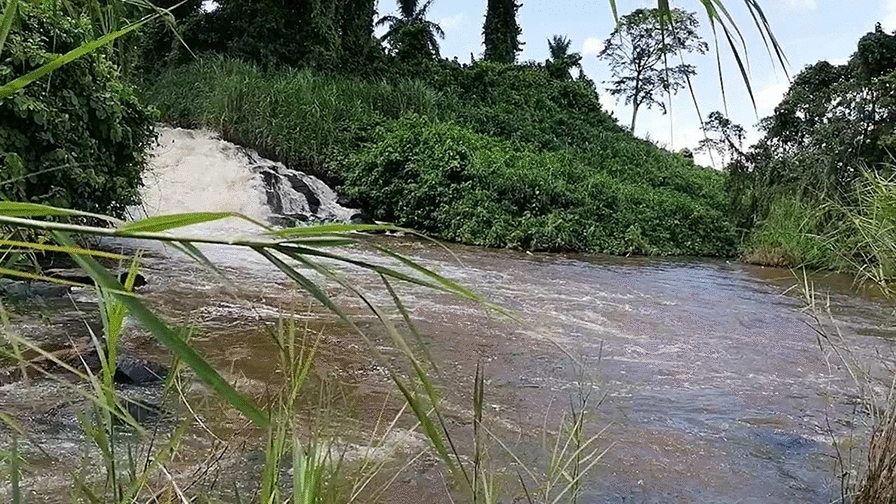

## Background

Onchocerciasis is a neglected tropical disease which manifests clinically as skin lesions and eye disease leading to blindness (also known as river blindness) [1]. It is caused by the filarial nematode *Onchocerca volvulus* transmitted by black flies (Diptera: Simuliidae) [1]. The black fly vectors breed in fast-flowing fresh water bodies, where they lay their eggs and develop into adult insects. Onchocerciasis is the world's second leading cause of preventable blindness. It is estimated that 99% of the 20.9 million individuals currently infected with *O. volvulus* live in Africa [1]. In addition to the clinical symptoms, onchocerciasis is a stigmatizing condition, which engenders significant psychosocial stress in the affected individuals [2, 3].

Foci of onchocerciasis endemicity were documented in Cameroon as early as the 1970s [4]. Various black fly species have been identified as potent *O. volvulus* vectors in Cameroon, with most of them belonging to the *Simulium damnosum* complex (*S. damnosum* sensu stricto, *S. sirbanum*, *S. yahense* and *S. squamosum*) [5]. Black fly breeding sites had been identified during previous studies along the Sanaga and Mbam rivers of Cameroon [6], accounting for the high onchocerciasis transmission in the surrounding villages.

Onchocerciasis elimination strategies in Cameroon rely mainly on annual community-directed treatment with ivermectin (CDTI) introduced since the late 1990s/early 2000s [7]. However, despite > 15 years of CDTI, onchocerciasis transmission is still ongoing in several parts of the country [7–9]. Besides causing skin and eye disease, high onchocerciasis transmission has also been associated with a high prevalence of epilepsy [10]. Indeed, recent surveys found a prevalence of epilepsy in onchocerciasis-endemic villages of Cameroon ranging from 2.5% to 7.8% [8, 9]. The most compelling evidence for the onchocerciasis-epilepsy association is provided by two cohort studies conducted in the Mbam Valley of Cameroon, where being infected with onchocerciasis during childhood was strongly associated with increased risk (up to 28-fold) of developing epilepsy later in life [11, 12]. Epilepsy is also known to be a stigmatizing condition [13], and its association with onchocerciasis obviously compounds the stigma experienced by persons with both conditions [14]. Fortunately, the implementation of effective onchocerciasis control measures would significantly reduce the epilepsy prevalence and incidence in affected communities over time, particularly in the 5–15-year age group [9, 15, 16].

In a bid to investigate the onchocerciasis prevalence in some Cameroonian villages and the impact of strengthening onchocerciasis elimination efforts on the incidence of epilepsy, a prospective study was initiated in the Ntui Health District in Cameroon in 2021 [17]. In the present article, we report the baseline findings regarding the onchocerciasis situation prior to implementing novel onchocerciasis control measures in the study villages.

## Methods

### Study setting

This research was conducted in rural communities of the Ntui Health District (Centre Region, Cameroon) from July 2021 to June 2022. The suburban town of Ntui is the headquarters for the Mbam et Kim division; it is located about 70 km north of the capital city Yaoundé and is surrounded by several villages. Agriculture is the main activity in the area with the production of both food and cash crops. The rural settlements located close to the Sanaga River constituted our study villages, and these included Essougli (04°23.850 N, 011°34.466 E), Nachtigal (04°22.672 N, 011°37.877 E), Ndjame (04°24.333 N, 011°38.258 E) and Ndowe (04°25.048, 011°42.835 E). Previous surveys conducted in 2009 and 2016 had confirmed that onchocerciasis was endemic in these villages, although the prevalence seemed to be decreasing with time [18, 19]. CDTI is usually conducted between June–July each year. However, due to COVID-19-related delays, the 2020 CDTI session was finally carried out in March 2021 in the Ntui Health District.

### Study procedures

Prior to conducting fieldwork, the research team visited the study area to identify villages located within 10 km of the Sanaga River. We met with the local authorities (village chiefs, health officers), explained the study objectives to them and obtained their consent. We then organized meetings with the community health workers for the Ntui Health District to plan for the conduct of the research project in their respective villages. The community health workers were given a few weeks to carry out massive sensitization and mobilization in their respective communities to inform the populations about the study and its potential benefits in the fight against onchocerciasis.

### Recruitment of participants and data collection

Following community engagement activities, the research team returned to the study villages and set up recruitment units in strategic locations (chief’s residence and/or primary schools). Village residents who were willing to participate came to the recruitment units where the research procedures were explained and their consent requested. Thereafter, a short questionnaire was administered to consenting participants to document their socio-demographic data (age, sex, profession, duration of stay in the village) and information about previous ivermectin use; all this information was obtained verbally from the respondents. A physical examination was conducted to assess the presence of dermatological lesions such as onchodermatitis [20]. The visual acuity of participants was measured using the tumbling E chart [21]. We also investigated a history of epilepsy among the participants using the recent operational definition of epilepsy provided by the International League Against Epilepsy (ILAE), that is, the occurrence of at least two unprovoked seizures > 24 h apart [22]. The diagnosis of epilepsy was made by the project physicians during fieldwork in the study villages.

Regarding sample collection from participants, we obtained a drop of blood by finger-pricking children aged 3–6 years to perform an Ov16 rapid diagnostic test (SD Bioline Onchocerciasis Ov16 IgG4, Standard Diagnostics, Gyeonggi-do, South Korea) for the presence of Ov16 antibodies as a proxy for onchocerciasis transmission in the community. For participants aged ≥ 5 years, two skin snips (one from each iliac crest region) were obtained using sterile 2-mm corneoscleral punches. The skin snips were placed in microtiter plates and incubated in isotonic saline for 24 h, after which they were examined under a microscope for the detection and quantification of *O. volvulus* microfilariae (mf) [23]. The average mf count for both skin snips from each participant was calculated. Microfilarial densities were expressed as mf/skin snip. The same experienced laboratory technician examined the skin snips from all study sites. All skin snip samples were obtained in July 2021, and Ov16 testing in children was conducted in July 2021 and March 2022. While the skin snip recruitment period in each of the villages of Essougli, Nachtigal and Ndjame lasted for at least 2 days, the research team was in Ndowe village only for 1 day because of logistical constraints; this explains why fewer participants were recruited from the latter site.

### Entomological study

The Sanaga River banks (specifically the portions adjoining the two riverside villages of Nachtigal and Essougli) were prospected to identify black fly breeding sites. These two sites were chosen because of their proximity to the Sanaga River and recent confirmation of prevalent cases of onchocerciasis [18], suggesting that transmission is ongoing. The river banks were explored by an experienced entomologist, and the surfaces of various submerged substrates (leaves, grasses, sticks and rocks) were carefully examined for the presence of black fly larvae and pupae. The black fly larvae identified during this exercise were rinsed and placed in Carnoy’s fixative for future cytotaxonomic studies [5]. We obtained the GPS coordinates of the breeding sites (where black fly larvae/pupae were identified) using a Garmin GPSMAP78 device.

Once the breeding sites were identified, we proceeded to collect adult black flies and measured biting rates via the human landing catch (HLC) technique described by Walsh et al. [24]. Black fly catching sites were set up at the river banks in close proximity with the breeding sites. Adult black flies were collected between 07:00 and 18:00 for 3 consecutive days each month, beginning in July 2021. In the present article, we will present baseline biting rate data for the first 12 months from July 2021 through June 2022. Black fly catchers in each site consisted of two individuals working alternate hours, who were recruited from the nearest village and trained in standard HLC methods. Locally made tubes [5] were used to aspirate black flies into an empty container every hour, after which hourly biting rates were measured by counting the number of black flies in each container. After counting, some adult black flies were dissected in the field to determine parity rates and to detect *Onchocerca* spp. larvae; the remaining black flies were preserved in 95% ethanol for subsequent pool screening.

### Sample size calculations

Sample size was calculated using Cochran’s formula, as follows: *N* = *p(1-p)z*^*2*^*/d*^*2*^ where *N* is the sample size, *p* is the prevalence of onchocerciasis in our study site (8.4% in villages of the Ntui Health District, based on a recent survey [18]), *z* is the decision variable at confidence of 95% (*z* = 1.96), and *d* is the sampling-related error risk (*d* = 0.05). The minimum sample size for the study was thus estimated at 119 participants.

### Ethical and administrative considerations

Prior to the study, ethical clearance was obtained from the institutional review board of the Cameroon Baptist Convention Health Board (Ref. IRB2021-03) as well as an approval from the Centre Regional Delegation of the Ministry of Public Health of Cameroon (Ref. 1156/AP/MINSANTE/SG/DRSPC); a research permit from the Ministry of Scientific Research and Innovation (Ref. 000,144/MINRESI/B00/C00/C10/C13), an ABS-Nagoya Protocol Prior Informed Consent (Ref. Decision No. 00016/D/MINEPDED/CNA) and ABS Permit No. 00013/MINEPDED/CAN/NP-ABS/ABS-FP) from the Ministry of Environment, Protection of Nature and Sustainable Development. The consent of the village authorities was obtained for the research project. Additionally, all participants signed an individual informed consent form, and the data obtained were treated confidentially.

### Data analysis

Data were analyzed in R version 4.0.2. Continuous variables were expressed as mean with standard deviation (SD) and compared across groups using the Mann-Whitney U test or the Kruskal-Wallis test as appropriate. Categorical data were expressed as percentages and compared using chi-squared tests. For multivariable analyses, we used skin snip positivity as dependent variable to investigate factors associated with *O. volvulus* infection in a multiple logistic regression model. In addition, we constructed a negative binomial Poisson regression model (because of the over-dispersion of the dependent variable) to investigate predictors of microfilarial density among infected participants; the superiority of this model over the ordinary Poisson regression model was confirmed by the Vuong test. For both models, covariates included participant age, sex and other variables which produced a *P*-value < 0.2 during univariate regression. The community microfilarial load (CMFL) for each village was calculated as the geometric mean of the individual mf load (to which the arbitrary value of 1 was added to have only non-zero values) in participants aged ≥ 20 years.

Regarding entomological data obtained from the two black fly catching points over a 12-month period, we used the biting rates for the 3 consecutive days of each month to calculate monthly biting rates as previously described [24]. Monthly transmission potentials were calculated using the method described by Walsh et al. [24], considering only data from the 1 day every month during which black flies were dissected in each village. As for annual transmission potentials, they were obtained by summing up the monthly transmission potentials for 12 consecutive months at each catching site. Similarly, annual biting rates were the sums of the monthly biting rates for each site. For all statistical analyses, two sided *P*-values < 0.05 were considered to be statistically significant.

## Results

A total of 460 participants were recruited from our four study villages, with 248 (53.9%) being males. The mean age was 32.1 (range 3–85) years. Most participants were farmers (52.0%, mostly adults) while 33.3% were students (the younger participants). The average duration of stay of participants in the study villages was 22.6 (range: 0.5–83) years. Among participants who were skin snipped, the overall prevalence of onchocerciasis (proportion of persons with positive skin snips) was 62/425 (14.6%; 95% confidence interval: 11.4–18.4). Participants from Ndjame village were older than those from the other villages (Table 1). The CMFL was 1.35, 1.24, 1.09 and 1.07 for the participants residing in Essougli, Nachtigal, Ndjame and Ndowe, respectively. Of the 373 participants who reported to have ever taken ivermectin in all the study villages, 202 (54.2%) confirmed that they received their most recent dose during the March 2021 CDTI session.

**Table 1:** Participant characteristics by village

	Essougli	Nachtigal	Ndjame	Ndowe	*P*-value
Distance from the river	0 km	0 km	≈8 km	≈5 km	
Number of participants: *n*	116	172	134	38	
Age, in years: mean (SD)	27.6 (20.6)	33.3 (22.4)	37.0 (22.6)	23.2 (17.7)	< 0.001*
Duration in village: mean (SD)	19.1 (18.4)	20.6 (20.6)	33.0 (23.0)	5.4 (6.3)	< 0.001*
Male gender: *n* (%)	58 (50.0%)	98 (57.0%)	66 (49.3%)	26 (68.4%)	0.124
Education level: *n* (%)					< 0.001*
None	14 (12.1%)	7 (4.1%)	16 (11.9%)	0 (0%)	
Primary	73 (62.9%)	112 (65.1%)	71 (53.0%)	30 (78.9%)	
Secondary	28 (24.1%)	39 (22.7%)	44 (32.8%)	8 (21.1%)	
University	1 (0.9%)	14 (8.1%)	3 (2.2%)	0 (0%)	
Previous ivermectin use: *n* (%)	92 (79.3%)	142 (82.6%)	118 (88.1%)	22 (57.9%)	< 0.001*
Number of previous ivermectin doses: mean (SD)	5.4 (4.4)	4.8 (3.9)	6.9 (4.5)	1.9 (2.4)	< 0.001*
Number of years since last ivermectin dose: mean (SD)^a^	1.7 (2.8)	2.3 (4.2)	1.1 (3.0)	0.7 (1.1)	< 0.001*
Tumbling E score: mean (SD)^a^	8.5 (2.2)	8.1 (2.4)	8.4 (2.3)	NA	0.292
Presence of skin lesion: *n* (%)	10 (8.6%)	19 (11.0%)	15 (11.2%)	4 (10.5%)	0.896
Positive skin snip: *n* (%)^b^	20/108 (18.5%)	24/159 (15.1%)	13/123 (10.6%)	5/35 (14.3%)	0.396
Microfilarial load: mean (SD)^b^	1.3 (4.9)	1.8 (9.2)	0.5 (3.8)	0.2 (0.8)	0.377
Epilepsy: *n* (%)	7 (4.1%)	7 (6.0%)	11 (8.21%)	0 (0%)	0.193

*SD* standard deviation, *NA* not available

^a^Eighty-seven missing data; ^b^35 missing data

^*^Statistically significant

We identified skin lesions in 48/460 (10.4%) participants. Among the latter, different skin lesions were represented as follows: papular lesions in 14 (29.2%), depigmentation in 13 (27.1%), lesions due to skin scratching in 10 (20.8%), “lizard skin” in 3 (6.3%) and desquamation in 2 (4.2%). The least common skin lesions with a single case each (frequency of 2.1%) included lymphoedema, subcutaneous nodule, vesicules, dermatomycosis, ulcerations and burn scars (in a participant who also had epilepsy). For study participants with available skin snip data (*n* = 425), we observed a trend: onchocerciasis prevalence increased with increasing age group; *P* = 0.187 (Table 2). Furthermore, when comparing *O. volvulus* infection among persons with epilepsy (PWE) (*n* = 24) and persons without epilepsy (*n* = 401), we found that those with epilepsy were more often skin snip positive (45.8% vs 12.7%; *χ*^*2*^ = 19.93, *df* = 1, *P* < 0.001) and had higher microfilarial loads (9.2 ± 22.0 vs 0.7 ± 3.5 mf/skin snip; Mann-Whitney U-test, *W* = 3115, *P* < 0.001) compared to their peers without epilepsy.

**Table 2:** Age-specific prevalence of onchocerciasis in the study population

Age group	*N*	Prevalence (95% CI)
3–5 years	11	0% (0.0–0.3)
6–10 years	60	8.3% (3.1–19.1)
11–19 years	76	11.8% (5.9–21.8)
20–29 years	57	19.3% (10.5–32.3)
30–39 years	49	14.3% (6.4–27.9)
40–49 years	47	10.6% (4.0–23.9)
≥ 50 years	125	20.0% (13.6–28.3)
Overall	62/425	14.6% (11.4–18.4)

*N* number of participants, *CI* confidence interval

### Factors associated with onchocercal infection

Among participants with skin snip data, the multiple logistic regression model revealed that having epilepsy and residing in Essougli (compared to Ndjame) were significantly associated with increased odds of being infected with *O. volvulus* (Table 3).

**Table 3:** Multiple logistic regression investigating factors associated with a positive skin snip

Covariates	Odd’s ratio (95% confidence interval)	*P*-value
Age	1.015 (0.991–1.038)	0.209
Male gender	1.473 (0.799–2.766)	0.220
Number of years since last ivermectin dose:	1.047 (0.966–1.124)	0.229
Having epilepsy	7.116 (2.433–20.599)	< 0.001*
Village: Essougli	Reference	
Village: Nachtigal	0.774 (0.373–1.626)	0.493
Village: Ndjame	0.371 (0.153–0.868)	0.024*
Village: Ndowe	0.872 (0.179–3.223)	0.849
Duration in the village (in years)	1.005 (0.983–1.028)	0.680

Number of participants included in model after removal of missing values, *n* = 367

*CI* confidence interval

^*^Statistically significant

We investigated predictors of the microfilarial load in infected participants. The negative binomial Poisson regression model revealed that having epilepsy was associated with increased *O. volvulus* parasitic load, while there was a trend of decreasing microfilarial load with increasing level of education (Table 4).

**Table 4:** Negative binomial Poisson regression analysis to investigate the factors associated with higher microfilarial load in infected participants

Covariates	Incidence risk ratio (95% confidence interval)	*P*-value
Age	0.987 (0.967–1.009)	0.181
Male gender	1.720 (0.751–3.904)	0.159
Having epilepsy	2.879 (1.187–7.979)	0.029*
No education	Reference	
Primary education	0.214 (0.024–1.088)	0.085
Secondary education	0.109 (0.012–0.571)	0.016*
University education	0.045 (0.002–5.067)	0.078
Village: Essougli	Reference	
Village: Nachtigal	2.400 (0.881–6.316)	0.069
Village: Ndjame	1.074 (0.370–3.257)	0.892
Village: Ndowe	0.597 (0.126–3.482)	0.511

Number of participants included in model after removal of missing values, *n* = 62

*CI* confidence interval

^*^Statistically significant

Although the variable “number of previous ivermectin doses” was not eligible to be included in any of the multivariable models (*P* > 0.2 in univariate analyses), we found that the median values for the number of previous ivermectin doses received by participants varied significantly across levels of education: 0 among those without education, four among those with primary education, seven for secondary education and six for the few participants with university education (Kruskal-Wallis test, *H* = 20.73, *df* = 3, *P* < 0.001).

### Entomological findings

**Fig. 1:**
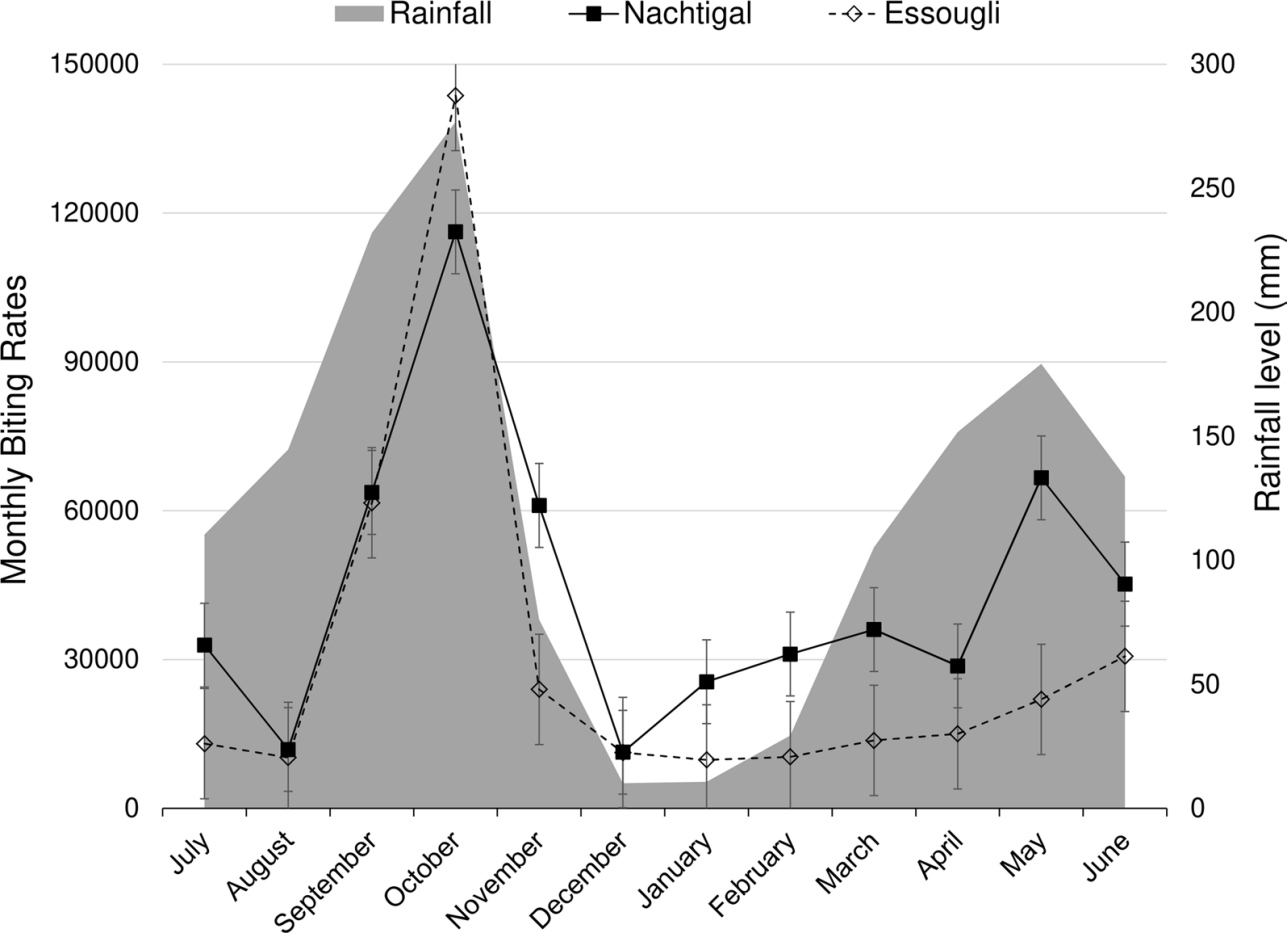
Evolution of monthly biting rates at the two catching sites. Rainfall levels are those from the neighboring Bafia Health District, obtained from Hendy et al. [5]

Breeding sites were identified in both Essougli (coordinates: N 4°23.156, E 11°33.131) and Nachtigal (coordinates: N 4°21.146, E 11°37.953). The number of black flies caught during the 3-day HLC period each month varied from 1096 (December) to 11,247 (October) in Nachtigal and from 947 (January) to 13,906 (October) in Essougli. The median monthly biting rates were higher in Nachtigal (34,477) compared to Essougli (14,366); Mann-Whitney U test, *W* = 35, *P* = 0.033. Monthly biting rates, parity, *O. volvulus* infection among black flies and transmission potentials for each catching site are summarized in Table 5. There were strong positive correlations between the monthly biting rates and the monthly rainfall levels in both Nachtigal (Spearman’s correlation coefficient, *rs* = 0.657, *P* = 0.024) and Essougli (Spearman rho = 0.685, *P* = 0.017); see Fig. 1. The annual biting rates in our black fly catching sites along the Sanaga River were 530,322 for Nachtigal and 365,404 for Essougli.

**Table 5:** Summary of black fly collection and dissection data from catches made at Nachtigal and Essougli

	July	August	September	October	November	December	January	February	March	April	May	June
Nachtigal												
Number of days	3	3	3	3	3	3	3	3	3	3	3	3
Total black fly catch	3184	1149	6370	11,247	6106	1096	2470	3333	3489	2871	6448	4522
Number dissected (%)	480 (15.1)	252 (21.9)	372 (5.8)	313 (2.8)	508 (8.3)	265 (24.2)	466 (18.9)	425 (12.8)	379 (10.9)	370(12.9)	276 (4.3)	318 (7.0)
Number parous (%)^a^	165 (34.4)	47 (18.7)	38 (10.2)	68 (21.7)	43 (8.5)	13 (4.9)	50 (10.7)	82 (19.3)	77 (20.3)	93 (25.1)	25 (9.1)	40 (12.6)
Number infected (%)^a^	1 (0.2)	3 (1.2)	2 (0.2)	0 (0)	4 (0.8)	0 (0)	2 (0.4)	1 (0.2)	1 (0.3)	0 (0)	0 (0)	0 (0)
Number with L3 (%)^a^	0 (0)	0 (0)	0 (0)	0 (0)	0 (0)	0 (0)	2 (0.4)^b^	0 (0)	0 (0)	0 (0)	0 (0)	0 (0)
Monthly biting rate	32,901	11,873	63,700	116,219	61,060	11,325	25,523	31,108	36,053	28,710	66,629	45,220
Monthly transmission potential	0	0	0	0	0	0	1479	0	0	0	0	0
Essougli												
Number of days	3	3	3	3	3	3	3	3	3	3	3	3
Total black fly catch	1262	993	6160	13,906	2400	1089	947	1114	1325	1504	2127	3066
Number dissected	399 (31.6)	167 (16.8)	410 (6.7)	321 (2.3)	381 (15.9)	231 (21.2)	215 (22.7)	182 (16.3)	213 (16.1)	331 (22)	312 (14.7)	372 (12.1)
Number (%) parous^a^	83 (20.8)	31 (18.6)	54 (13.2)	49 (15.3)	44 (11.5)	20 (8.7)	14 (6.5)	13 (7.1)	48 (22.5)	45 (13.6)	31 (9.9)	40 (10.8)
Number infected (%)^a^	2 (0.5)	3 (1.8)	0 (0)	1 (0.3)	2 (0.5)	1 (0.4)	2 (0.9)	0 (0)	1 (0.5)	2 (0.6)	1 (0.3)	0 (0)
Number (%) with L3^a^	0 (0)	1 (0.6)	0 (0)	0 (0)	0 (0)	0 (0)	0 (0)	0 (0)	1 (0.5)	0 (0)	1 (0.3)	0 (0)
Monthly biting rate	13,041	10,261	61,600	143,695	24,000	11,253	9786	10,397	13,692	15,040	21,979	30,660
Monthly transmission potential	0	50	0	0	0	0	0	0	58	0	74	0

^a^Calculations based only on dissected black flies, not on total number of black flies caught

^b^Of these two infected black flies, one had 4 L3 larvae and the other had 14 L3 larvae (total of 18 L3 larvae in the heads of the two black flies)

Overall, 7586 black flies were dissected during the 12 months with 5 (0.07%) found to be infected with the L3 larval form of *O. volvulus* in their heads. These L3-infected black flies were distributed as follows: 2/4052 (0.05%) in Nachtigal and 3/3534 (0.08%) in Essougli. The annual transmission potentials in our study sites were 1479 for Nachtigal and 182 for Essougli.

### Ov16 seroprevalence in children aged 3–6 years

Of the 123 children between the ages of 3 and 6 years who were tested for Ov16 antibodies, the rapid diagnostic test results were positive in 8, giving a seroprevalence of 6.5% (95% CI 3.1–12.8). There was no significant difference in Ov16 seroprevalence across villages (Fisher exact test with more than two categories, *P* = 0.467) or across ages (Fisher exact test with more than two categories, *P* = 0.540).

## Discussion

Our study provides up-to-date data on the epidemiology of onchocerciasis in the Ntui Health District. A prevalence of *O. volvulus* infection of 14.6% was found, which is slightly higher than the 10.7% obtained in 2016 [18], which itself was a drop from a 26.4% prevalence in 2009 [19]. This sinusoidal evolution of onchocerciasis prevalence in the study area could be due to inconstant CDTI therapeutic coverage over the years. For instance, only 54% of our study participants with a history of ivermectin use had taken the drug in 2021, representing sub-optimal therapeutic coverage of ivermectin distribution. Interestingly, villages bordering the Sanaga River (Essougli and Nachtigal) had higher onchocerciasis prevalence and CMFL compared to other villages, although this was not statistically significant. This trend concurs with previous findings in Cameroon and Ethiopia [4, 25, 26], demonstrating increasing onchocerciasis burden with decreasing distance from the river.

Similar to previous reports [27], we found that the prevalence of onchocerciasis increased with age in our population although this was not statistically significant. However, none of our participants aged ≤ 5 years was infected, in contrast to the study of Nana-Djeunga et al. who found 6/169 (3.6%) of children < 5 years with positive skin snips in Mbangassina village still within the Ntui Health District [28]. This can be explained by the fact that the Mbangassina area is crisscrossed by both the Sanaga River and its tributary, the Mbam River, which probably increases the black fly population thereby increasing onchocerciasis transmission. Comparing with the Mbangassina data, we note that our study villages are not the most endemic hotspots in the district.

Contrary to what was reported by previous studies [25, 29], we did not find an inverse relationship between epilepsy prevalence and the distance of the village from the river (see Table 1). This could be due to our enrollment approach, which required that participants move to the recruitment site, which may be less efficient in detecting persons with epilepsy (PWE) compared to the gold-standard door-to-door strategy. In addition, it is likely that several PWE in the study villages were not enrolled into the study because of stigma and/or poor health conditions, which hindered them from reporting at the recruitment sites. That notwithstanding, the few PWE who did participate were more *O. volvulus*-infected than participants without epilepsy, supporting the existence of an association between onchocerciasis and epilepsy [10]. This hypothesis is further strengthened by the multivariable models, which showed that having *O. volvulus* infection and especially higher microfilarial loads was significantly associated with epilepsy, in line with previous findings from studies in Cameroon and abroad [11, 12, 25, 30].

Another notable finding from the multivariable models was the association between the educational level and microfilarial load among infected participants, in that a higher education level led to reduced infection intensity. Additionally, persons who were more educated were likely to have taken ivermectin more times in the past. Taken together, these two observations underscore the importance of educating the community to improve onchocerciasis practices, including adherence to CDTI [31, 32]. It is likely that more educated individuals adopt healthier habits regarding exposure to infected black flies. In the past, other authors have also reported an association between educational status and onchocerciasis infection [26]. Indeed, mathematical modeling studies reveal that education is a key intervention which contributes to controlling the dynamics of onchocerciasis transmission in endemic settings [33].

The annual biting rates along the Sanaga river observed in this study were a little lower compared to those in villages close to the Mbam River [5]. However, monthly observations revealed that during some months, there are more black flies along the Sanaga River than the Mbam River. In that regard, although the peak monthly biting rates for both the Sanaga and Mbam rivers were experienced during the month of October (month of maximum rainfall; see Fig. 1), the values for villages along the Sanaga river banks (in the present study: 143,695 and 116,219) were more than double those reported along the Mbam River banks (73,418 and 28,623) [5]. However, the lower biting rates in the Mbam river valley are compensated by higher black fly infection rates. A 12-month entomological study in two villages bordering the Mbam River banks showed that 17/7166 (0.2%) of the dissected black flies had infective *O. volvulus* L3 larvae in their heads [34], while the present study found only 5/7586 (0.07%) L3-infected black flies in the two villages on the Sanaga riverside. Therefore, the annual transmission potentials along the Mbam River (4488 and 2360) [5, 34] were higher than in the present study along the Sanaga river (1479 and 182), suggesting that more intense onchocerciasis transmission occurs in the Mbam river valley. Of note, previous entomological research already established that only the Sanaga River and its main tributary, the Mbam River, harbor black flies responsible for transmitting onchocerciasis, while their smaller tributaries are free of vectors [6]. Therefore, implementing vector control activities in these two rivers will further plummet the transmission potentials and accelerate onchocerciasis elimination prospects.

One possible explanation for the lower infection rates observed among black flies and humans during our study (compared to findings from the Mbam river) is the fact that CDTI was conducted in the study villages in March 2021, barely 4 months before the start of our survey. Following treatment with ivermectin, skin repopulation by *O. volvulus* mf has been shown to slowly resume within 3 to 6 months, but not reaching the initial infection intensity [35, 36]. Additionally, previous research in endemic communities in Cameroon has suggested that in villages with > 20 years of CDTI as is the case with our study sites, there is a lower mf clearance and higher repopulation rate after treatment with ivermectin [37]. One can reasonably argue that, despite the sub-optimal coverage, the ivermectin treatment had lowered the community microfilarial load in the study villages such that the probability of a black fly taking up mf during a blood meal would also be low. A prospective follow-up for *O. volvulus* detection in black flies and humans every month until the next CDTI session would provide more insight into these infection dynamics.

We observed that Ov16 seroprevalence among the 3–6 year olds in our study was low (6.5%) compared to 40% in a similar age group in South Sudan [38], 15–63% among children aged 6–10 years in villages of the Lekie division downstream of the Sanaga river in Cameroon (co-endemic with loiasis) [39] and 42.4–55.4% among older children aged 7–10 years in villages of the Mbam Valley in Cameroon [8, 9]. The low Ov16 seroprevalence in our study sites should be interpreted with caution as it could be influenced by the low age group we tested and our enrollment procedure at a fixed spot in the village which may have limited access for some children to participate, especially those living very close to the river. It could also be that the Ov16 antibody titers in the younger Cameroonians are relatively low, rendering the rapid diagnostic tests less sensitive in the 3–6 years age group. In summary, while *O. volvulus* transmission may be relatively lower in our study villages compared to other sites, our data show that the Ntui Health District is still endemic for onchocerciasis and would benefit from optimal measures to accelerate onchocerciasis elimination.

A major limitation of this work was the fact that recruitment was not done using a door-to-door approach, thereby introducing some selection bias for estimating the true prevalence of onchocerciasis and epilepsy in the study villages. However, the fact that participants from different villages took part in the study provides some perspective regarding the spread of onchocerciasis across the different communities in the Ntui Health District.

## Conclusion

Our study has documented ongoing transmission of onchocerciasis in villages of the Ntui Health District alongside a sub-optimal ivermectin coverage among the village residents. Despite high biting rates, black fly infection rates (by microscopy) were rather low resulting in low transmission potentials. In addition to the reduced infection rates brought about by CDTI, interventions to reduce the high black fly biting rates could drastically limit *O. volvulus* transmission in these communities. Mindful of the disease burden caused by onchocerciasis and the numerous socioeconomic benefits that its elimination could bring in these communities [40], it is expedient that control strategies be strengthened to achieve the elimination goals sooner rather than later. It is to this end that the novel “Slash and Clear” vector control strategy will be deployed in the study area [17].

## Data Availability

The datasets for our study findings are available from the corresponding author on reasonable request.
